# The Upside of Slackers

**DOI:** 10.1371/journal.pbio.1000487

**Published:** 2010-09-14

**Authors:** Robin Meadows

**Affiliations:** Freelance Science Writer, Fairfield, California, United States of America

**Figure pbio-1000487-g001:**
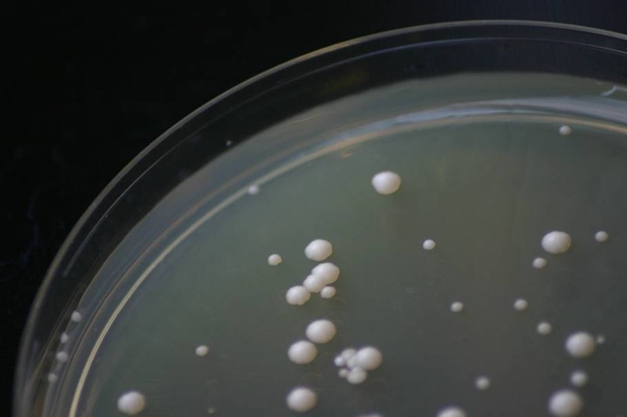
Invertase-producing (large colonies) and non-producing (small colonies) yeast competing on agar initially containing sucrose as the sole carbon source. Image: Eric Miller and Duncan Greig.

[Fig pbio-1000487-g001]Are slackers a drag on the rest of us? It makes sense because those who produce (“co-operators”) benefit everyone, whereas those who don't produce (“cheats”) get a free ride. In yeast, for instance, most strains are co-operators, secreting an enzyme called invertase that converts sucrose into the more efficiently metabolized glucose. However, about a tenth of strains are cheats that do not secrete invertase, thus enjoying the benefits of glucose without incurring the costs of production. Classic theory holds that cheating comes at the expense of society as a whole, making populations composed entirely of co-operators the most fit. But this may not be true, according to new research reported in this issue of *PLoS Biology* by Laurence Hurst and Ivana Gudelj.

The researchers' counterintuitive finding reflects their novel approach. Rather than starting with the classic theory and then looking for experimental support, Hurst and Gudelj began with the experimental finding that a mix of cheats and co-operators yielded the biggest yeast populations. To understand this unexpected result, they developed a model accounting for factors including yeast growth rates, invertase production and sugar use. The researchers validated the model partly by showing that it accurately predicted that population growth (a measure of fitness) peaked when there were cheats as well as co-operators.

The model predicted that cheats benefit populations when three conditions are met. One is that yeast use glucose more efficiently when it is scarce. When glucose is abundant, uptake rises but efficiency of use drops due to metabolic constraints, thus curbing the growth rate of yeast relative to resource availability. To test the necessity of this rate-efficiency tradeoff, the researchers took advantage of the fact that it effectively disappears when yeast is grown at very low sucrose levels. As expected, limiting sucrose restored the classic result that populations are most fit when everyone co-operates, showing that cheats benefit populations when there is a rate-efficiency tradeoff. By damping overall glucose production, cheats make this resource used more efficiently by the population as a whole.

Another condition is that yeast cannot tell how much sucrose remains to be converted to glucose, making co-operators produce invertase inefficiently. Yeast keeps secreting this enzyme even after all the sucrose is gone because invertase production depends on how much glucose is left. To test the necessity of imperfect information about sucrose levels, the researcher used a model assuming that invertase production depended on sucrose rather than glucose levels. Again, this restored the classic result, verifying that cheats benefit populations that lack accurate information about resource availability.

The final condition is that instead of being homogenous, the population is structured so that cheats and co-operators are clumped by strain type, ensuring that the latter get more of the glucose they produce. To test the necessity of population structure, the researchers grew yeast in shaken flasks to mix cheats and co-operators as thoroughly as possible. This also restored the classic result, confirming that cheats benefit populations that are structured.

Why the discrepancy between this new model and the well-established theory to the contrary? The classic result that co-operators are best for society arises from models such as the snowdrift game, where individuals are stuck in snow and can either co-operate by shoveling it or cheat by just using the cleared paths. In yeast, making invertase is the equivalent of shoveling snow and providing glucose is the equivalent of clearing paths.

While the snowdrift game has been used to show that yeast populations are most fit when everyone co-operates, the researchers point out that this model makes assumptions that are not true for yeast. The snowdrift game assumes that the benefit of production remains constant, whereas yeast uses glucose less efficiently when it's abundant. The game also assumes that the total cost is fixed because there is only so much snow to be shoveled. However, the cost of producing invertase is not fixed because yeast keep making this enzyme even after all the sucrose is gone.

So-called co-operators and cheats are common in societies from microorganisms to people, and game theory is used to inform economic and social policy. But people are far more complex than yeast and even this microorganism may be too complex to be neatly divided into co-operators and cheats. We all have different strengths and a slacker in one context may be a contributing member of society in another.

That caveat aside, the conditions under which cheats contribute to the greater good may be widespread. The researchers argue that populations are likely to be structured, scanty information is likely to make co-operator production outstrip the need, and tradeoffs between resource availability and efficiency of use are common. Indeed, people waste less food during famines.

This thought-provoking work questions the conventional wisdom that co-operation is fundamental to the well-being of groups as well as the conventional approach to studying co-operation, and helps explain the diversity of strategies in social interactions. It will be illuminating to see if cheats are also good for yeast populations in the wild as well as for groups of other species.

**MacLean RC, Fuentes-Hernandez A, Greig D, Hurst LD, Gudelj I (2010) A Mixture of “Cheats” and “Co-Operators” Can Enable Maximal Group Benefit. doi:10.1371/journal.pbio.1000486**

